# Optical Polarization Division Multiplexing Transmission System Based on Simplified Twin-SSB Modulation

**DOI:** 10.3390/s22207700

**Published:** 2022-10-11

**Authors:** Ye Zhou, Jun Ming, Leilei Wang, Dongyan Wu, Li Zhao, Jiangnan Xiao

**Affiliations:** 1 School of Optical Electrical and Computer Engineer, University of Shanghai for Science and Technology, Shanghai 200093, China; 2 School of Information Science and Technology, Fudan University, Shanghai 200433, China

**Keywords:** PDM, Twin-SSB, system complexity, spectral efficiency, de-mapping algorithm

## Abstract

Optical twin-single sideband (Twin-SSB) modulation, due to the left sideband (LSB) and right sideband (RSB) signal carrying individual data, has become an attractive technique in fiber transmission because it satisfies the demand of the explosive increase in data traffic. This paper focuses on reducing the complexity of Twin-SSB system and further enhancing the spectral efficiency by proposing a polarization division multiplexing (PDM) Twin-SSB modulation scheme. LSB and RSB signals are extracted using de-mapping algorithm instead of optical bandpass filters (OBPFs) to reduce system complexity. To further improve spectral efficiency, PDM is employed to meet the polarization multiplexing transmission and achieve a higher transmission capacity. Based on the PDM Twin-SSB system, the LSB is 3-arr phase-shift-keying (3PSK) modulated, while RSB is quadrature phase-shift keying (QPSK) modulated. We simulated that the bit error ratio (BER) performance of LSB and RSB of X-polarization (X-Pol) and Y-polarization (Y-Pol) at 8-Gbaud, 10-Gbaud, 12-Gbaud, 14-Gbaud, and 16-Gbaud in the case of back-to-back (BTB) and 2 km standard single-mode fiber (SSMF) transmission. The simulation results verify the effectiveness and practical feasibility of the proposed PDM Twin-SSB scheme for future short-distance transmission owing to low cost, simplified structure, low algorithm complexity, and high data transmission capacity.

## 1. Introduction

With the rapid development of information services, such as 5G, virtual reality (VR), cloud computing, and other new services, such as smart homes and autonomous driving, access network traffic is increasing explosively. The potential capacity demand imposes challenging burdens on optical transmission communication. To improve the capacity of optical transmission systems, a variety of advanced system architectures using novel modulation formats have been reported, including optical single sideband (SSB) modulation technology with direct detection (DD) [1,2,3,4,5,6]. These schemes have various advantages over conventional double sideband (DSB) modulation. This is due to the fact that this scheme is not only free from the dispersion-induced radio frequency power fading observed in the transmission of DSB signals but is also capable of doubling the spectral efficiency by halving the spectral width of DSB signals [7,8,9,10]. In addition, efficient transmission power is expected. In these schemes, however, there are two serious shortcomings in the optical SSB system: the first is that the signal-signal beat interference (SSBI) induced by the square-law detection of the photodetector (PD) significantly degrades the system performance, and the second is that the partial bandwidth of the transmitter digital-to-analog converters (DACs) is wasted because the optical SSB signal only carries one sideband information. To improve spectral efficiency, Twin-single sideband (Twin-SSB), an efficient modulation technique evolving from the SSB modulations [11,12], has recently been studied for bandwidth-economic radio communication systems [13,14,15,16,17,18,19,20,21,22,23], in which two groups of independent signals are modulated onto the LSB and the RSB [24]. Compared with the optical SSB system, the optical Twin-SSB system has many advantages. Particularly, it nearly doubles the transmission capacity when compared to the optical SSB scheme because the LSB and RSB carry independent data information. As a result, the Twin-SSB modulation can theoretically eliminate the dispersion-induced power fading and double the spectral efficiency due to the SSB modulation for the independent signals and fully utilizing the redundant sideband. Ref. [25] proposes a modulation technique called Twin-SSB. To generate Twin-SSB signals, a commercially available dual-driver Mach–Zehnder modular (DDMZM) is used. Two groups of independent signals are modulated onto LSB and RSB. At the receiver side, two steep OBPFs and PDs are employed for signal detections, compared with the conventional SSB signal, which has the potential to double the spectral efficiency. Ref. [26] proposes a simple 2 × 2 multiple-input multiple-output (MIMO) optical-wireless integration system, in which optical independent SSB modulation enabled by an in-phase-quadrature modulator (IQM), instead of DDMZM, is used to assist the simultaneous generation of Twin-SSB signals. However, all of these schemes require two steep OBPFs to separate LSB and RSB signals at the receiver side, resulting in a complex and unstable system structure. In addition, the conventional Twin-SSB system scheme still has some shortcomings, the most serious of which is their crosstalk. The reason is that the OBPFs cannot completely separate LSB and RSB signals, resulting in two optical signals with residual optical signals of the other optical signal; moreover, the input power of two independent-sideband signals is unbalanced [27,28,29,30,31]. As a result of these factors, system performance deteriorates. To mitigate this problem, a guard band between two independent sidebands and optical carrier is required, which results in spectral waste. Ref. [13] uses an optical IQM to optimize optical carrier-to-signal power ratio (CSPR), which further improves the receiver sensitivity of spectrally efficient guard-band DD optical orthogonal frequency-division multiplexing with Twin-SSB modulation technique. Ref. [14] proposes a Twin-SSB suppressed carrier (Twin-SSBSC) which can be modulated using a commercially available DP-QPSK modulator. The signals on the LSB and RSB can be demultiplexed by simple electrical operations including Hilbert transforms without requiring steep OBPFs. Dispersion tolerant characteristics and error vector magnitude (EVM) performance was investigated by numerical simulation. Ref. [11] proposes a spectrally efficient Twin-SSB single carrier system based on MIMO processing. In C-band transmission, 264 Gb/s per polarization over 80 km SSMF with a net bit rate of 220 Gb/s using 88 GSa/s DACs is experimentally demonstrated with KK receiver. To the best of our knowledge, almost all studies on Twin-SSB require two OBPFs and PDs to separate and detect signals at the receiver side. This not only increases system complexity and cost but also deteriorates receiver performance due to hardware imperfections, particularly the non-ideal property of the OBPF. Furthermore, in most previous Twin-SSB works, only one optical polarization is utilized. As is known to all, however, the optical polarization division multiplexing (PDM) technique is a promising method for spectral-efficient optical transmission, which can be used to double the system capacity and spectral efficiency [32,33,34,35,36]. Thus, the optical PDM signal is adopted in this paper to improve system capacity and spectral efficiency.

In this study, we propose a low complexity PDM Twin-SSB modulation scheme, in which LSB and RSB signals are detected by one single-ended PD and extracted by de-mapping algorithm. Compared with the conventional Twin-SSB scheme, our proposed scheme needs no OBPFs to split LSB and RSB signals and only requires one single-ended PD and ADC at the receiver side. Simultaneously, the optical PDM technology is adopted to realize a higher data capacity and spectral efficiency. By an orchestra of these techniques, the carrier is modulated on LSB and RSB signals in X-polarization (X-Pol) and Y-polarization (Y-Pol). The polarization separation and Twin-SSB signal generation are implemented using a cascaded polarization beam splitter (PBS) and IQM, which is also helpful for the carrier suppression of the opposite polarization. At the receiver side, the signal is split into two branches, and two branches are detected without OBPFs filtering. The Y-Pol interference in the X-Pol or X-Pol interference in the Y-Pol is mitigated by constant modulus algorithm (CMA) algorithm. After digital signal processing (DSP), LSB and RSB signal in X-Pol and Y-Pol are separated by de-mapping algorithm. We first simulate the PDM Twin-SSB system BER performance at 8-Gbaud, 10-Gbaud, 12-Gbaud, 14-Gbaud, and 16-Gbaud in BTB case. We then demonstrate the 2-km SSMF transmission with a BER below the hard-decision forward-error-correction (HD-FEC) threshold of 3.8 × 10^−3^. It is shown that our proposed PDM Twin-SSB scheme based on de-mapping algorithm is feasible, and that the system capacity can be approximately doubled compared to the single optical polarization system.

## 2. Principle

### 2.1. Principle of Twin-SSB Signal Generation

**Figure 1:**
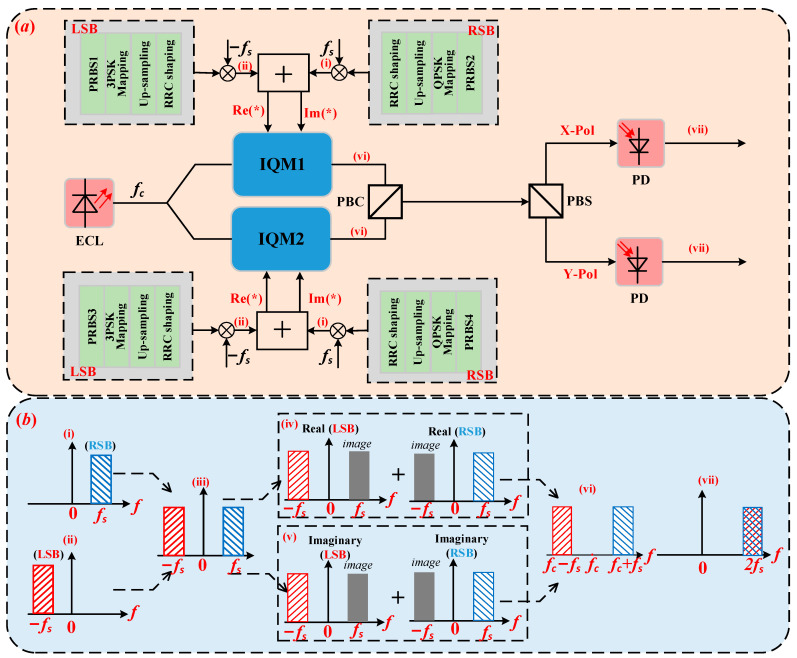
(**a**): The generation scheme of the PDM Twin-SSB. (**b**) Single polarization spectrum diagram of Twin-SSB signal generation. inset (**i**): Schematic diagrams of RSB signal; inset (**ii**): Schematic diagrams of LSB signal; inset (**iii**): Schematic diagrams of Twin-SSB signal; inset (**iv**): Schematic diagrams of I-path digital domain signal; inset (**v**): Schematic diagrams of Q-path digital domain signal; inset (**vi**): Schematic diagrams of Twin-SSB signal through I/Q modulator; inset (**vii**): Schematic diagrams of received digital domain signal. ECL: external cavity laser; IQM: in-phase and quadrature modulator; PD: photodetector; PBC: polarization beam combiner; PBS: polarization beam splitter.

A schematic diagram of the PDM Twin-SSB signal generation is depicted in Figure 1. At the transmitter, first, four sets of independent pseudo-random binary sequences (PRBSs), which are denoted by PRBS1, PRBS2, PRBS3, and PRBS4, are 3PSK and QPSK mapped and filtered by root raised cosine (RRC) filter to generate four independent signals represented by $S_{X,L}(t)$, $S_{X,R}(t)$, $S_{Y,L}(t)$, and $S_{Y,R}(t)$, respectively, where $S_{X(Y),L}(t)=A_{X(Y),L}\exp [j\theta_{X(Y),L}(t)]$, $S_{X(Y),R}(t)=A_{X(Y),R}\exp [j\theta_{X(Y),R}(t)]$, $\theta_{X(Y),L}$, and $\theta_{X(Y),R}$ are the phase information of signal carried on the LSB and RSB signal of X-Pol and Y-Pol, $A_{X(Y),L}$ and $A_{X(Y),R}$ are signal amplitude of LSB and RSB signal of X-Pol and Y-Pol, respectively. $S_{X(Y),L}(t)$ corresponds to LSB signal in X-Pol or Y-Pol, while $S_{X(Y),R}(t)$ corresponds to RSB signal in X-Pol or Y-Pol respectively.

After that, signal $S_{X,L}(t),S_{X,R}(t),S_{Y,L}(t)$, and $S_{Y,R}(t)$ are upconverted using complex sinusoidal radio-frequency (RF) sources with frequencies $-f_{s}$ and $f_{s}$. The LSB and RSB signal of X-Pol and Y-Pol can be expressed as:

(1) $$E_{X,L}(t)=A_{X,L}\exp [-j2\pi f_{X,L}t+j\theta_{X,L}(t)]$$

(2) $$E_{X,R}(t)=A_{X,R}\exp [j2\pi f_{X,R}t+j\theta_{X,R}(t)]$$

(3) $$E_{Y,L}(t)=A_{Y,L}\exp [-j2\pi f_{Y,L}t+j\theta_{Y,L}(t)]$$

(4) $$E_{Y,R}(t)=A_{Y,R}\exp [j2\pi f_{Y,R}t+j\theta_{Y,R}(t)]$$

The spectrograms of the LSB signal and the RSB signal are shown in Figure 1(b(i,ii)), respectively. Then, the four independent SSB signals are combined to obtain Twin-SSB signal, which can be expressed as follows:

(5) $$E_{X,\text{Twin-SSB}}(t)=E_{X,L}(t)+E_{X,R}(t)$$

(6) $$E_{Y,\text{Twin-SSB}}(t)=E_{Y,L}(t)+E_{Y,R}(t)$$

where $E_{X,\text{Twin-SSB}}(t)$ and $E_{Y,\text{Twin-SSB}}(t)$ are Twin-SSB signal of X-Pol and Y-Pol, respectively. Consequently, the real part and imaginary part of the Twin-SSB signal of X-Pol are used to drive IQM1, while the real part and imaginary part of the Twin-SSB signal of Y-Pol are used to drive IQM2. For the optical IQM, the real and imaginary parts of the signal are uploaded into the two input ports of the IQM. The continuous wave (CW) optical signal with the center frequency $f_{c}$ emitted from an external cavity laser (ECL) is divided into two branches by a polarization maintaining optical coupler (OC) with the same power. One is used as the optical input of IQM1, and the other is used as the optical input of IQM2. Both IQM1 and IQM2 work at the lowest bias point. Thus, the output signal can be expressed as:

(7) $$E_{\mathrm{IQM}1}(t)\approx E_{CW}(t) [A_{X,L}J_{-1}(m)\exp\left(-j2\pi f_{s}t+j\theta_{X,L}\left(t\right)\right)+A_{X,R}J_{1}(m)\exp\left(j2\pi f_{s}t+j\theta_{X,R}\left(t\right)\right)]$$

(8) $$E_{\mathrm{IQM}2}(t)\approx E_{CW}(t) [A_{Y,L}J_{-1}(m)\exp\left(-j2\pi f_{s}t+j\theta_{Y,L}\left(t\right)\right)+A_{Y,R}J_{1}(m)\exp\left(j2\pi f_{s}t+j\theta_{Y,R}\left(t\right)\right)]$$

where $J_{-1}(.)$ and $J_{1}(.)$ are Bessel functions of the first class, and m is the modulation coefficients. Then, the outputs of IQM1 and IQM2 are coupled together by a polarization beam combiner (PBC). Figure 1(b(vi)) shows the output of the IQM.

After SSMF transmission, the received signal is first separated into the two orthogonal polarizations (X-Pol and Y-Pol) by using a single polarization beam splitter (PBS) and a finely adjusted polarization controller (PC). Then, signal of each polarization is directly fed into PD. Note that dropped OBPFs reduce complexity of Twin-SSB system, where the left and right sidebands carrying the data and the center carrier will beat each other in the PD. Taking the X-Pol as an example, by setting the modulation coefficients to 1, the photocurrent generated can be expressed as:

(9) $$i_{X,\mathrm{PD}}(t)=R{\left|E_{IQM1}(t)\right|}^{2}\approx R{\left|E_{CW}(t)\right|}^{2}\left\{\begin{array}{l} \frac{1}{2}A^{2}{}_{X,L}+\frac{1}{2}A^{2}{}_{X,R}\\ +A_{X,L}A_{X,R}\cos\left(\theta_{X,L}(t)-\theta_{X,R}(t)\right)\\ +A_{X,L}A_{X,R}\cos\left(2\pi \cdot \left(2f_{s}\right)t+(\theta_{X,L}(t)-\theta_{X,R}(t))\right)\\ +\frac{1}{2}A^{2}{}_{X,L}\cos(2\pi \cdot \left(2f_{s}\right)t+2\theta_{X,L}\left(t\right))\\ +\frac{1}{2}A^{2}{}_{X,R}\cos(2\pi \cdot \left(2f_{s}\right)t+2\theta_{X,R}\left(t\right)) \end{array}\right\}$$

where *R* represents PD responsivity. Figure 1(b(vii)) is the output of the PD. The output of the PD contains the direct current part, the baseband signal, the desired sideband signal, and the crosstalk. In Equation (9), the first term is direct component which can be filtered. The second term is the baseband signals which can be neglected. The third term is desired signal obtained from the beating of modulated LSB and RSB signal locating at $2f_{s}$. The fourth and fifth term are generated from the beating of LSB and RSB whose center frequency also locating at $2f_{s}$, which will induce crosstalk. As for Y-polarization, the process is the same. By adding equalization algorithm, the crosstalk can be mitigated to an acceptance value.

### 2.2. Principle of Twin-SSB Signal Detection Based on a Single PD

Since the received signal is an intermediate frequency signal, down-conversion is applied to attain baseband signal. After DSP algorithm, quadrature demodulation is performed to obtain the in-phase and quadrature components. According to Equation (9), owing to the signal amplitude being a constant and possible to be eliminated by normalization in the subsequent DSP, we mainly consider the relationship between $S_{X}(t)$ and the values of $\theta_{X,L}(t)$ and $\theta_{X,R}(t)$. Taking the X-Pol signal as an example, Table 1 shows the values of $S_{X}(t)$ corresponding to different values of $\theta_{X,L}(t)$ and $\theta_{X,R}(t)$. the LSB signal is 3PSK modulated, where the phase $\theta_{X,L}(t)$ value of the LSB signal is $\left(0,\;2\pi /3,\;4\pi /3\right)$, and the RSB signal is QPSK modulated, where the phase $\theta_{X,R}(t)$ value of the RSB signal is $\left(\pi /4,\;3\pi /4,\;5\pi /4,\;7\pi /4\right)$.

From Table 1, it can be found that the values of $S_{X}(t)$ corresponding to different values of $\theta_{X,L}(t)$ and $\theta_{X,R}(t)$. Table 1 shows the value of $S_{X}(t)$ is $\left(\pi /12,\;3\pi /12,\;5\pi /12,\;7\pi /12,\;9\pi /12,\;11\pi /12,\;13\pi /12,\;15\pi /12,\;17\pi /12,\;19\pi /12,\;21\pi /12,\;23\pi /12\right)$. That is, the signal $S_{X}(t)$ is a 12PSK signal, and each constellation point corresponds to a combination of $\theta_{X,L}(t)$ and $\theta_{X,R}(t)$. For example, consider the constellation point with phase π/12 of $S_{X}(t)$. The constellation phase is the sum of LSB 3PSK 4π/3 and RSB QPSK 3π/4 constellation, which indicates that, at the receiver side, phase π/12 of $S_{X}(t)$ signal is separated as phase 4π/3 of 3PSK and phase 3π/4 QPSK signal constellation using the simplified offline de-mapping algorithm.

## 3. Simulation Settings and Results

### 3.1. Simulation Settings

**Figure 2:**
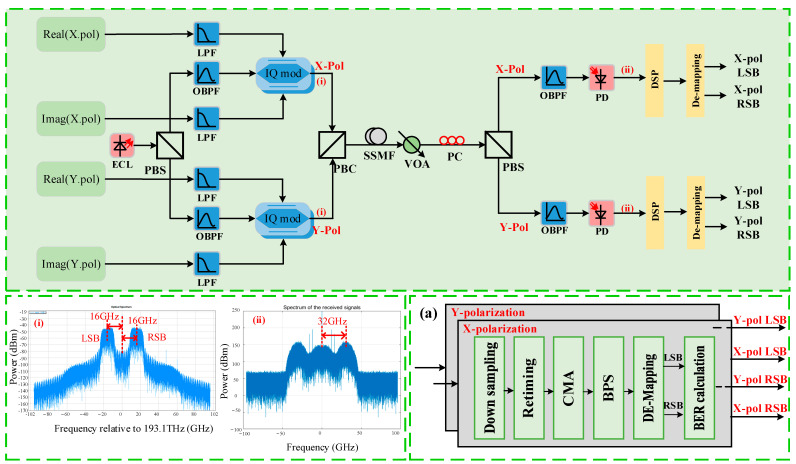
Simulation setup and DSP of the simplified PDM Twin-SSB system; inset (**i**): Optical spectra of Twin-SSB signal after IQM; inset (**ii**): Spectrum of received electrical signal via PD; inset (**a**) DSP diagram of the PDM- Twin-SSB signal at the receiver side. LPF: low-pass filter; PBC: polarization beam combiner; VOA: variable optical attenuator; PC: polarization controller; PBS: polarization beam splitter; OBPF: optical bandpass filter.

To verify the PDM Twin-SSB transmission performance, the simulation setup is described in detail in Figure 2. At the transmitter, the Twin-SSB signals are modulated with a light-wave at 1548.706 nm generated from an external cavity laser (ECL) with <100 kHz linewidth and 16 dBm output power. X-Pol signal is generated as follows: two sets of PRBSs are generated using offline programming. Here, the PRBS length is 2^14^. LSB adopts 3PSK modulation, while RSB adopts QPSK modulation. Next, independent SSB signals are up-sampled and digitally shaped using a root raised cosine (RRC) with a roll-off factor of 0.01. Similarly, another two sets of PRBSs are generated for transmission of the Y-Pol state. Corresponding to 16 GHz carrier spacing, LSB adopting 3PSK modulation is linearly converted located at a carrier frequency of −16 GHz, respectively, while RSB signal adopting QPSK modulation is linearly converted located at a carrier frequency of 16 GHz. After up-conversion and serial conversion, the PDM Twin-SSB signals of X-Pol and Y-Pol are loaded into ports of IQM. Figure 2i shows the measured output optical spectrum of the IQM with two independent LSB and RSB sideband spaced by 16-GHz from the central optical carrier. In our simulation, we adjust the three DC biases of the IQM using two steps. In the first step, we adjust the three DC biases to ensure that the two MZM have a minimum output optical power and the PM has a π/2 phase shift. In the second step, we appropriately adjust the DC biases of two MZM while fixing the DC bias of the PM. The generated Twin-SSB signals are combined via a PBC and fed into the fiber link.

After SSMF transmission, a variable optical attenuator (VOA) is employed to adjust the received optical power. Then, the received signal is separated into the two branches orthogonal polarizations (X-Pol and Y-Pol) by PBS, and then each polarization is simultaneously detected by one single-ended PD. Since the output of the IQM cannot guarantee the fixed polarization state, the polarization controller (PC) is required before PBS to match the polarization state of the received signal. Subsequently, LSB and RSB signals of X-Pol and Y-Pol is upconverted by one single-ended PD to 32-GHz electrical signal, respectively. The measured spectrum is shown in Figure 2ii. To achieve the simulation results close to the experimental results, we add an OBPF with 45-GHz in front of one single-ended PD to limit the bandwidth of PD. Noting that the polarization of the fiber after PBC is arbitrary, and the X-Pol and Y-Pol component at the output port of the PBC contains a mixture of the data, which is simultaneously encoded on the X-Pol and Y-Pol at the transmitter.

The captured signals are offline processed, and the receiver side DSP diagram is shown in Figure 2a. The signal down-conversion is also achieved at first. After that, retiming is executed in order. The 21-tap T/2 spaced CMA equalizer is followed to realize polarization demultiplexing and remove the crosstalk at the same antenna polarization. Blind Phase Search (BPS) algorithm is used to further compensate the phase noise, and then recover the LSB and RSB signals of each polarization using de-mapping algorithm. Finally, the BER calculation is followed.

For a DD system, the optical CSPR should be optimized owing to the SSBI mitigation. we first optimize CSPR so that this PDM Twin-SSB system work at the optimal condition. The CSPR is adjusted by fixing the optical carrier power before loading into the IQM and changing driving signal power for the IQM at the transmitter DSP. When the CSPR is too low, the crosstalk induced by the beating term of modulated signals from LSB and RSB cannot be neglected. A high CSPR can efficiently reduce the crosstalk induced by the beating of the LSB and RSB signals. However, the optical signal-to noise ratio (OSNR) of signals will also be decreased with the increasing CSPR.

**Figure 3:**
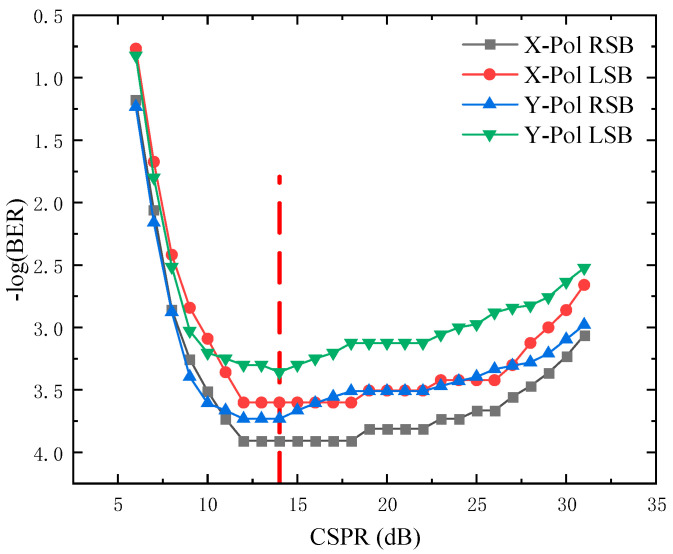
BER versus the CSPR for 8-Gbaud LSB and RSB of X-Pol and Y-Pol at the 16 dBm received optical power BTB transmission scenarios, respectively.

Figure 3 shows the measured BER performance at different CSPR conditions for 8-Gbaud signals versus the CSPR from 5 dB to 30 dB at the −16 dBm received optical power. From 5 dB to 13 dB, the BER performance is improved with the increasing of CSPR. However, when the CSPR is larger than 14 dB, the BER performance begins to deteriorate, so the optimal CSPR is 14 dB.

### 3.2. Results

**Figure 4:**
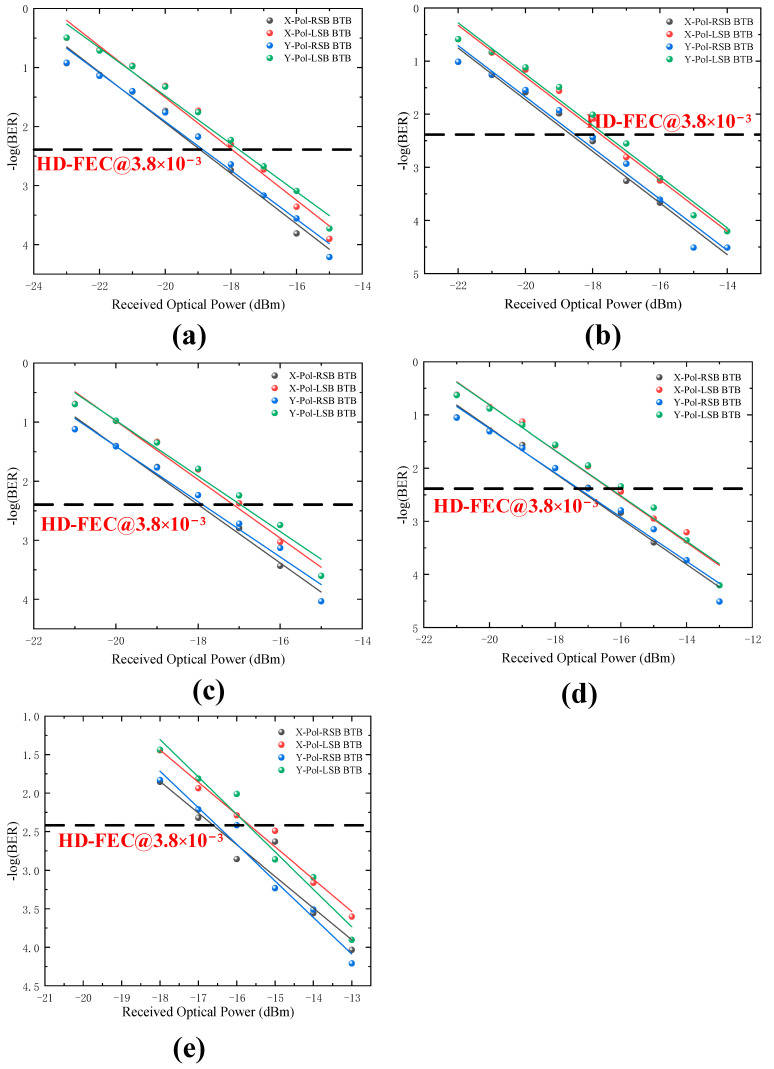
BER versus the received optical power for different baud rates at BTB transmission scenario. (**a**) 8-Gbaud, (**b**) 10-Gbaud, (**c**) 12-Gbaud, (**d**) 14-Gbaud, (**e**) 16-Gbaud.

Figure 4a–e show the measured LSB and RSB signals BER of X-Pol and Y-Pol versus the received optical power for 8-Gbaud,10-Gbaud, 12-Gbaud, 14-Gbaud, and 16-Gbaud at BTB transmission scenario. We can conclude that no matter which baud rate is used (i.e., 8-, 10-, 12-, 14-, or 16-Gbaud), the BER of LSB and RSB in X-Pol and Y-Pol is lower than the HD-FEC threshold of 3.8 × 10^−3^. For BTB transmission, higher baud rate can be expected, and thus higher spectral efficiency can be seen. However, at the threshold of 3.8 × 10^−3^, compared to the 8-Gbaud, the 16-Gbaud case shows about 2-dB power penalties. The degradation of BER performance is mainly due to the higher baud rates bringing larger interference.

**Figure 5:**
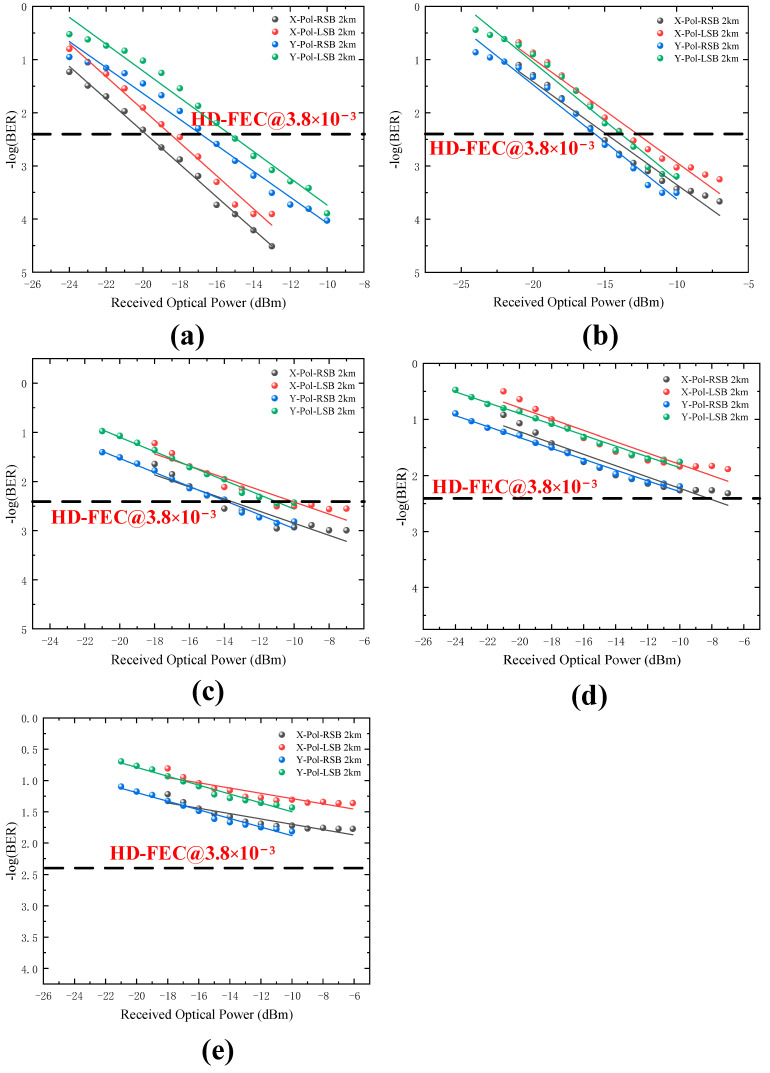
BER versus the received optical power for different baud rates at 2 km transmission scenario. (**a**) 8-Gbaud, (**b**) 10-Gbaud, (**c**) 12-Gbaud, (**d**) 14-Gbaud, (**e**) 16-Gbaud.

Figure 5a–e show PDM Twin-SSB signal BER performance at 2-km SSMF transmission scenarios with 8-Gbaud, 10-Gbaud, 12-Gbaud, 14-Gbaud, and 16-Gbaud rate cases. When baud rate is lower than 14-Gbaud, the BER can reach below the HD-FEC threshold of 3.8 × 10^−3^. In Figure 5e, it can be found that the BER cannot reach below the HD-FEC threshold of 3.8 × 10^−3^ after 2-km SSMF channel transmission. The BER performance of the is significantly reduced compared to the 16-Gbaud BTB transmission. From Figure 4 and Figure 5, increasing the received optical power may improve the BER performance.

## 4. Conclusions

In this study, we propose a low complexity PDM Twin-SSB modulation scheme which features a receiver front-end with reduced hardware comprising a single-ended PD. We also improved spectral efficiency by exploiting the PDM technique. The feasibility of the PDM Twin-SSB scheme is verified by simulation experiment, where 16 GHz 8-Gbaud, 10-Gbaud, 12-Gbaud, 14-Gbaud, and 16-Gbaud mapped LSB 3PSK and RSB QPSK of X-Pol and Y-Pol BTB and transmission over 2 km SSMF are achieved with the aggregate BER below the HD-FEC threshold of 3.8 × 10^−3^. The results show that our proposed simplified PDM Twin-SSB scheme is a promising candidate for future short-distance optical transmission.

## Figures and Tables

**Table 1:** The relationship between $S_{X}(t)$ and the value of $\theta_{X,L}(t)$ and $\theta_{X,R}(t)$.

	$\theta_{X,R}(t)$	π/4	3π/4	5π/4	7π/4
$\theta_{X,L}(t)$					
0		exp(j3π/12)	exp(j9π/12)	exp(j15π/12)	exp(j21π/12)
2π/3		exp(j11π/12)	exp(j17π/12)	exp(j23π/12)	exp(j5π/12)
4π/3		exp(j19π/12)	exp(jπ/12)	exp(j7π/12)	exp(j13π/12)

## Data Availability

Not applicable.
